# Clinical research informatics (CRI): overview over new tools and services

**DOI:** 10.1186/2043-9113-5-S1-S1

**Published:** 2015-05-22

**Authors:** Wolfgang Kuchinke, Töresin Karakoyun

**Affiliations:** 1 Heinrich-Heine University, University Hospital, 40225 Düsseldorf, Germany

## Introduction

In many ESFRI infrastructures [1] and EU projects, tools to support data sharing and biomedical research are being developed. At the Clinical Research Informatics Solutions Day (May 26-27, 2014 at Heinrich-Heine University, Düsseldorf, Germany) new tool developments, their interoperability and sustainability issues in connection with EU projects, like BioMedBridges, BBMRI, ECRIN, EATRIS, ELIXIR, TRANSFoRm, p-medicine and EHR4CR, were discussed. These discussions resulted in three main insights: first, biomedical research increasingly needs the sharing of data from electronic health records (EHR), reinforcing the necessity for trust and privacy protection; second, requirements are increasing for semantic integration of data from very heterogeneous resources (clinical records, biosamples, images, genetic repositories); third, big data and health care data will be used more intensively to determine patient subgroups, for better patient recruitment and to improve trial feasibility [2]. The graphic method developed by the TRANSFoRm project to describe and analyse data privacy frameworks using a zone model [3] addresses this development and may be useful for all projects that have to deal with sharing care and research data.

Tools and services that support similar functionalities were presented in joint workshops allowing developers and users to interact and compare tools. CRI has chosen this format to give developers and users opportunities to collaborate with each other to improve usability of tools. In separate workshops tools for clinical data management (OpenClinica, ObTiMA, VISTA), for bridging experimental and clinical research data (tranSMART, MOLGENIS, i2b2), for the reuse of EHR data for clinical research (Feasibility Service, Patient Screening Tool, Recruitment and Feasibility Tools); for advanced clinical data management (TRANSFoRm eCRF, mobile eHealth Solution), for imaging in clinical research (XNAT, DoctorEye), and for biobanking in clinical research (p-BioSPRE, Biobanking Catalogue, BBMRI Catalogue) were presented and discussed.

## Overview

Recently, EU projects, like TRANSFoRm, p-medicine, EHR4CR and BioMedBridges, have created advanced clinical research information systems consisting of sets of tools / services embedded in IT platforms that enable data protection and data provenance tracing. These new tools are able to assemble data from heterogeneous sources to answer complex research questions, and support research process workflows to meet the needs of translational and personalized medical research. The overall impression of the workshops is that data collection has evolved from simple access and input of data to data linking, enrichment with metadata, integration of EHR data, mobile eHealth data, biobank data and data warehousing.
